# Dysregulation of Podocyte BK Channels and Nephrosis: Effects of Circulating Factors and Auxiliary β4 Subunits

**DOI:** 10.3390/cells14010022

**Published:** 2024-12-30

**Authors:** Eun Young Kim, Patrycja Rachubik, Stuart E. Dryer

**Affiliations:** 1 Department of Biology and Biochemistry, University of Houston, Houston, TX 77204, USA; ekim8@central.uh.edu (E.Y.K.); p.rachubik@gmail.com (P.R.); 2 Laboratory of Molecular and Cellular Nephrology, Mossakowski Medical Research Institute, Polish Academy of Sciences, 02-106 Gdansk, Poland; 3 Department of Biomedical Sciences, Tilman J. Fertitta Family College of Medicine, University of Houston, Houston, TX 77204, USA

**Keywords:** podocyte, calcium, TRPC6, BK channel, nephrosis, TNFα, circulating factor

## Abstract

Podocytes express large-conductance Ca^2+^-activated K^+^ channels (BK channels) and at least two different pore-forming KCa1.1 subunit C-terminal splice variants, known as VEDEC and EMVYR, along with auxiliary β and γ subunits. Podocyte KCa1.1 subunits interact directly with TRPC6 channels and BK channels become active in response to Ca^2+^ influx through TRPC6. Here, we confirmed that Ca^2+^ influx through TRPC channels is reduced following the blockade of BK channels by paxilline. The overall abundance of KCa1.1 subunits, as well as that of β4 and γ3 subunits, were increased in glomeruli isolated from Sprague Dawley rats during chronic puromycin aminonucleoside (PAN) nephrosis. Exposing cultured mouse podocytes for 24 h to recombinant TNFα, a circulating factor implicated in pediatric nephrotic syndromes, did not affect the total abundance of KCa1.1, but did evoke significant increases in both β4 and γ3. However, TNFα evoked a marked increase in the surface abundance of KCa1.1 subunits, similar to that of its previously reported effects on TRPC6 channels. The effect of TNFα on the surface expression of KCa1.1 was eliminated following siRNA knockdown of the β4 subunits, suggesting a role for this subunit in KCa1.1 trafficking to the cell surface. By contrast, treating podocytes with suPAR did not affect the total or surface expression of KCa1.1. The coordinated activation of KCa1.1 channels may promote Ca^2+^ influx through TRPC channels during normal and abnormal podocyte function by maintaining a membrane potential that allows for the efficient permeation of divalent cations through TRPC pores.

## 1. Introduction

The sustained hyperactivation of canonical transient receptor potential-6 channels (TRPC6) leads to glomerular disease in humans and animals [1]. Patients with various gain-of-function mutations in TRPC6 develop severe focal and segmental glomerulosclerosis (FSGS), e.g., [2,3,4]. The podocyte-specific overexpression of certain mutant TRPC6 channels in mice, in particular M131T, results in podocyte foot process effacement, proteinuria, and glomerulosclerosis [5]. Conversely, the global inactivation of TRPC6 is partially reno-protective in animal models of FSGS, for example, in the chronic PAN nephrosis model in rats [6]; it also protects the glomerular compartment in autoimmune glomerulonephritis [7].

TRPC6 is a non-selective cationic channel that is permeable to Ca^2+^ under certain conditions; there is evidence that podocyte dysfunction is a consequence of sustained TRPC6-mediated Ca^2+^ overload. Thus, the influx of Ca^2+^ through TRPC6 results in the activation of multiple downstream targets, including calcineurin [8,9,10] and the Ca^2+^-dependent protease calpain [11], resulting in changes in gene expression in the cell body, altered cytoskeletal organization in foot processes, and eventually to cell death [9,10]. TRPC6 channels in podocytes form complexes with several other proteins in slit diaphragm domains, including nephrin [12], podocin [3,13], and NADPH oxidases [14], all of which have important consequences for the gating and trafficking of TRPC6 [1]. In addition, endogenous podocyte TRPC6 channels interact directly with large-conductance Ca^2+^-activated K^+^ channels (also known as BK, Maxi-K, or Slo1 channels) [15].

Because TRPC6 and other TRPC channels are non-selective cation channels, their activation will always cause cell depolarization in physiological conditions; this effect will occur regardless of whether or not there is Ca^2+^ influx through its pores. However, the permeability of the TRPC6 pore to divalent cations is strongly dependent on membrane potential [16]. While TRPC6 channels are permeable to monovalent cations at all membrane potentials, their permeability to Ca^2+^ is markedly reduced as a result of membrane depolarization within a physiological range [16]. An important consequence of this is that TRPC6 channel activation by itself will result in a decrease in the Ca^2+^ permeability of these channels, thereby causing them to behave as monovalent cation channels [16]. This effect is in addition to the decrease in electrodiffusive driving force that occurs during depolarization and that would decrease Ca^2+^ influx through all TRPC channels, including TRPC3, TRPC5 or TRPC6. Therefore, in order for TRPC activation to drive Ca^2+^-dependent cascades inside podocytes during normal physiological responses or during disease processes, there needs to be a mechanism to reduce the magnitude of the depolarization that would normally occur as a result of TRPC activation.

One potential mechanism for this is the coordinated coupling of BK channels to TRPC activation. BK channels are activated by Ca^2+^ binding to specific sites on cytoplasmic-facing domains of the four principal pore-forming subunits, which are known as KCa1.1 subunits [17]. We have previously shown that endogenous TRPC6 channels of podocytes co-immunoprecipitate with KCa1.1 proteins [15] and that the activation of TRPC6 results in activation of BK channels in podocytes [1]. The KCa1.1 subunits of BK channels occur in many different splice variants, including three different variants at the carboxy terminus [18]. Two of these carboxy terminal variants are found in mouse podocytes; we should note that one of these, the so-called VEDEC variant, exhibits highly regulated trafficking to the cell surface that is dependent on interactions with other proteins, including nephrin and TRPC6 [18,19].

The properties of BK channels are also determined by auxiliary subunits including β and γ subunits [17]. Auxiliary β and γ subunits are not necessary to form a functional BK channel in a heterologous expression system, i.e., they do not form part of the pore; however, they extensively modify various gating properties, including the Ca^2+^ and voltage dependence and kinetics of activation and deactivation. It is likely that all, or nearly all, endogenously expressed BK channels in various cell types contain auxiliary subunits. The BK channels of podocytes are notable for their very slow activation kinetics and their partial resistance to inhibition by iberiotoxin, which can be explained by the presence of β4 subunits [17,20,21]. Mouse podocytes also express the γ3 subunit (sometimes known as LRRC55) which markedly enhances BK activation at membrane potentials in the physiological range of podocytes [22].

One consequence of these observations is that cellular changes that facilitate the activation of BK channels on the cell surface would be expected to enhance Ca^2+^ influx through TRPC6 and other TRPC channels under normal physiological conditions. This could occur through changes in the overall abundance and/or location of KCa1.1, β4, or γ3. Indeed, an important previous study has shown that increases in the γ3 subunit occur in patients with FSGS, diabetic nephropathy, and membranous nephropathy, and that BK channel activation can enhance TRPC6-mediated Ca^2+^ influx in podocytes [22]. In the present study, we have examined whether various experimental conditions that model glomerular diseases are associated with changes in the abundance or distribution of BK channel subunits, with a special focus on two of the soluble circulating factors that have been suggested to play a role in primary FSGS.

## 2. Materials and Methods

### 2.1. Chronic PAN Nephrosis

These protocols were approved by the University of Houston Institutional Animal Care and Use Committee (protocol number 201800033) following NIH guidelines. The protocol used here was similar to those described in detail previously [6]. Briefly, male and female Sprague Dawley rats (100–150 g) were administered a single i.p. injection of PAN (Sigma-Aldrich, St. Louis, MO, USA) dissolved in 0.9% sterile saline at a dose of 200 mg/kg. Control animals received only saline at the same time. The animals were carefully monitored. Thirty days after the injection, blood and urine were sampled, the animals were euthanized, and the kidneys were excised. Urine albumin was measured using a commercial ELISA assay (Exocell Inc., Philadelphia, PA, USA), and creatinine in plasma and single-spot samples of urine were measured using an ELISA assay from Abcam (Boston, MA, USA). A portion of the renal cortex of one kidney was removed and reserved for biochemical analyses. Glomeruli were isolated using standard sieving protocols [13,23]. The rest of the kidney was used for histology.

### 2.2. Histology

Portions of the renal cortex were immersion-fixed, embedded in paraffin, and 4-μm serial sections were stained by periodic acid–Schiff’s (PAS) and Masson’s trichrome methods and by immunohistochemistry. These samples were evaluated by an observer blind to the treatment group. For each animal, the glomerular score (GS) in PAS-stained sections was graded using a scale of 0–4, in which 0 was assigned to normal glomeruli; 1 denoted glomeruli with mesangial expansion; 2 denoted glomeruli in which the sclerosis encompassed less than 50% of the glomerulus; 3 denoted glomeruli with lesions encompassing 50–75% of the glomerulus; and 4 denoted glomeruli with lesions encompassing more than 75% of the glomerulus or fully collapsed glomeruli. GS was evaluated in at least 25 glomeruli from each animal representing at least 10 sections. The resulting scores were averaged to obtain a mean value for each animal. Statistical analysis was then carried out on the mean GS values from each group of animals (control and PAN-treated).

### 2.3. Cell Culture

An immortalized mouse podocyte cell line (MPC-5 cells, originally obtained from Dr. Peter Mundel), was propagated and maintained at 33 °C, as described previously [13,19]. The passage number of the cells used in these experiments was <15. Differentiation of the podocytes was induced by removal of the γ-interferon and a temperature switch to 37 °C for 14 days. Immortalized podocytes cultured in this way express nephrin and podocin after differentiation.

### 2.4. Fluorescent Ca^2+^ Imaging in Cultured Podocytes

Quantitative fluorescence Ca^2+^ imaging was carried out using the indicator dye Calybryte-520™ (AAT Bioquest, Pleasanton, CA, USA), which has Ca^2+^-dependent excitation and emission properties similar to Fluo-4 [24]. Calbryte-520 AM-ester was prepared at a final concentration of 5 µM in a loading solution containing 0.04% Pluronic F127 (AAT Bioquest), according to the manufacturer’s instructions. Cells were loaded for 60 min in a CO_2_ incubator at 37 °C before analysis. Sequential images of emissions at 520 nm were obtained using a fluorescence microscope during excitation at 488 nm. TRPC channels in the podocytes were activated by superfusion of a hypoosmotic stretch solution (70% of control), which induces robust and sustained activation of TRPC6 channels in podocytes, as described in several previous studies from our laboratory [13,25,26]. We chose this stimulus because it produces a robust and highly reproducible activation of TRPC6 in immortalized mouse podocytes. Application of the hypoosmotic stretch was carried out in the control cells, as well as in cells pretreated for 10 min with the TRPC6 inhibitor 4-[[(1R,2R)-2-[(3R)-3-amino-1-piperidinyl]-2,3-dihydro-1H-inden-1-yl]oxy]-3-chlorobenzonitrile dihydrochloride (SAR-7334) (100 nM) or the BK channel inhibitor paxilline (20 µM).

### 2.5. Immunoblot Analysis and Cell-Surface Biotinylation Assays

For the immunoblot analyses, proteins in the podocyte lysates or isolated glomeruli were separated by SDS-PAGE on 10% gels and transferred to membranes. The blots were incubated with a primary antibody overnight at 4 °C, followed by horseradish peroxidase (HRP)-conjugated secondary antibody for 1 h at room temperature. Proteins were visualized using a chemiluminescent substrate. The antibodies against KCa1.1 and the BK channel β4 subunit were obtained from Alomone Labs (Jerusalem, Israel). An antibody against the γ3 subunit was obtained from Novus Biologicals (Centennial, CO, USA). All the primary antibodies were used at a dilution of 1:1000. To assess changes in the expression of KCa1.1 on the cell surface, podocytes subjected to various treatments described later were treated with a membrane-impermeable biotinylation reagent, sulfo-*N*-hydroxy-succinimidobiotin (1 mg/mL in PBS buffer; Pierce Biotechnology, Rockford, IL, USA) for 1 h. The reaction was stopped, and biotinylated proteins on the cell surface were recovered from cell lysates by incubation with immobilized streptavidin–agarose beads (Pierce Biotechnology). A sample of the initial cell lysate was used for analysis of the total cellular KCa1.1. These samples were separated on SDS–polyacrylamide gel electrophoresis; proteins were quantified by immunoblot analysis, as described above. This allowed for calculation of the ratio of KCa1.1 on the surface to the total KCa1.1.

### 2.6. siRNA Knockdown

For the transient, small, interfering RNA (siRNA) experiments, siRNA targeting the β4 subunit of BK channels (sc-146370) and a non-targeted control siRNA (sc-37007) were purchased from Santa Cruz Biotechnology (Santa Cruz, CA, USA) and transfected into the podocytes using Oligofectamine™ (ThermoFisher Scientific, Waltham, MA, USA) in a serum-reduced medium, according to the manufacturer’s instructions. The effectiveness of knockdown was assessed by immunoblot, as described above.

### 2.7. Drugs, Recombinant Proteins, and Other Reagents

Recombinant TNFα and suPAR were obtained from R&D Systems (Minneapolis, MN, USA). SAR-7334 and paxilline were obtained from BioTechne (Minneapolis, MN, USA).

### 2.8. Statistical Methods and Quantitative Analyses

Data were analyzed by Student’s unpaired *t*-test or one-way ANOVA and Tukey’s honestly significant difference post hoc test using public-access computational tools (http://www.vassarstats.net (accessed on 10 December 2024) with *p* < 0.05 regarded as significant. The data in bar graphs are presented as fold changes relative to the lowest value observed in a control group and are presented as mean ± SD.

## 3. Results

**Figure 1:**
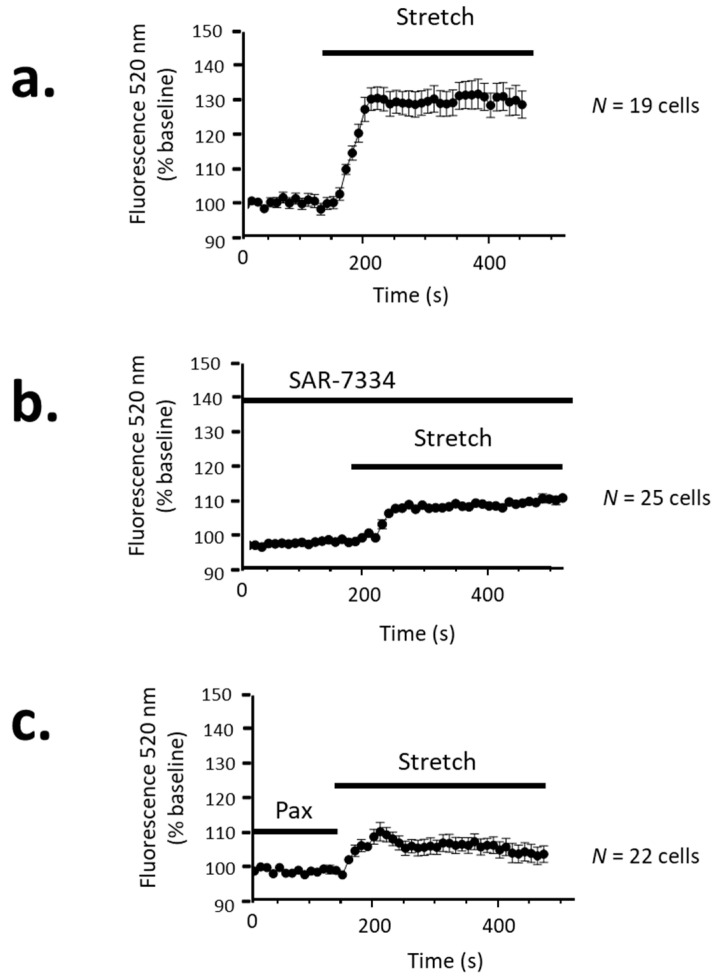
Quantitative fluorescence measurement of cytosolic free Ca^2+^ in immortalized podocytes in response to a hypoosmotic stretch stimulus known to cause robust and sustained activation of TRPC6. The Ca^2+^ signal was measured using the indicator dye Calybryte-520™: (**a**) a hypoosmotic stretch stimulus caused a marked increase in cytosolic free Ca^2+^ that was maintained for as long as the stretch stimulus was maintained, as we have reported previously for TRPC6 channels [13]; (**b**) pre-exposure for 10 min to the TRPC6 blocker SAR-7334 (100 nM) caused a marked reduction in the amplitude of this signal; and (**c**) a similar reduction was observed in podocytes pre-treated for 10 min with the BK channel inhibitor paxilline (20 μM). Points represent means amplitudes relative to baseline fluorescence, error bars represent SEM, and the number of cells examined is indicated. The inhibition by paxilline suggests that BK channels allow for enhanced Ca^2+^ influx through TRPC6.

In the initial experiments, we examined whether the presence of functional BK channels can alter TRPC-mediated Ca^2+^ influx into cultured mouse podocytes, as reported earlier [22]. In these experiments, we utilized the indicator dye Calybryte-520™, which has Ca^2+^-dependent excitation and emission properties similar to Fluo-4 [24], but which yielded a stronger signal in podocytes. We activated podocyte TRPC6 using a hypoosmotic stretch stimulus that we have described in detail in a number of previous patch clamp analyses [13,25,26]. Specifically, exposing the podocytes to a solution with an osmotic strength of 70% of the control causes a robust activation of TRPC6 that can be sustained for as long as the stimulus is maintained [13], and which is completely blocked by the TRPC6 inhibitor SAR-7334 [1]. We observed a similar pattern with Ca^2+^ imaging, specifically, a marked increase in cytosolic free Ca^2+^ that was sustained for as long as the stimulus was maintained, at least up to several minutes (Figure 1a). However, pretreatment with 100 nM SAR-7334 for 10 min caused a marked reduction in the amplitude of the stretch-evoked Ca^2+^ signal (Figure 1b). At this concentration, SAR-7334 inhibits TRPC6 and TRPC3, but would not affect other TRP channels [27]. It should be noted that the endogenous TRPC of podocytes could include TRPC3 and TRPC6 heteromers. These experiments do not reveal the nature of the SAR-7334-resistant signal; however, we have previously shown that this drug causes a complete inhibition of podocyte TRPC6 currents at this concentration [1]. To assess the role of BK channels, we utilized 20 µM paxilline, which was shown in our previous work to cause the complete and irreversible inhibition of macroscopic BK currents in podocytes [19]. This drug would not affect other ion channels at this concentration [28]. In cells pretreated with paxilline for 10 min, we observed a marked reduction in stretch-evoked Ca^2+^ influx, comparable to that observed with SAR-7334 (Figure 1c). This observation is consistent with the hypothesis that the coordinated activation of Ca^2+^-dependent BK channels of podocytes [1] enables TRPC6 channels to sustain Ca^2+^ influx; this confirms the earlier observations and conclusions of Hu et al. [22]

**Figure 2:**
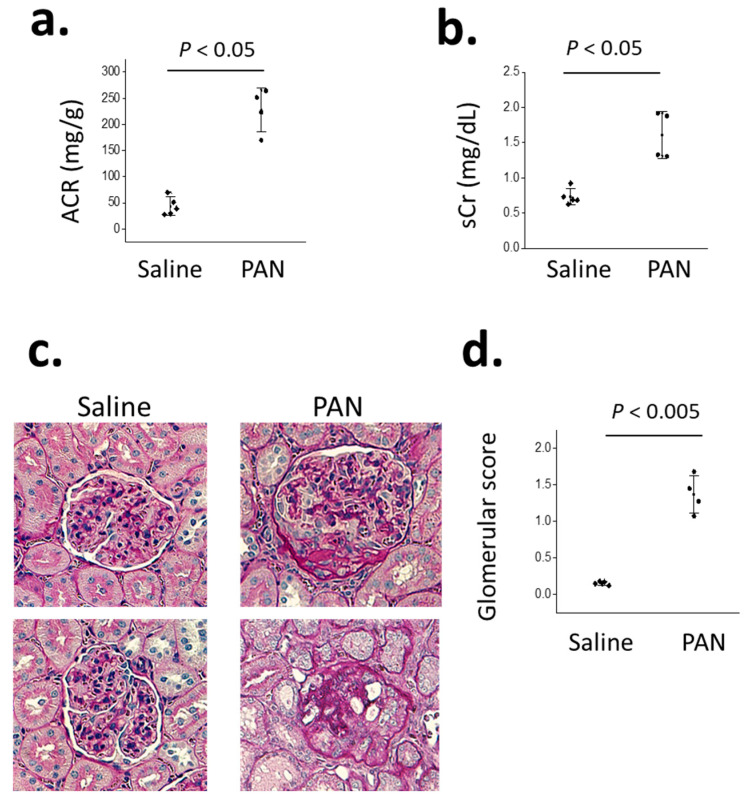
Evidence of kidney disease in Sprague Dawley rats 30-days after treatment with PAN or saline, as indicated. This experimental design is the chronic PAN nephrosis model: (**a**) increase in spot urine albumin:creatinine ratios (ACR) following PAN treatment; (**b**) increase in serum creatinine in PAN-treated rats compared to the control, indicating a decrease in the glomerular filtration rate; (**c**) glomerulosclerosis in PAN-treated rats compared to the saline-treated controls shown in representative PAS-stained section; and (**d**) mean glomerular score in saline- and PAN-treated rats based on the analyses of PAS-stained sections by an observer blind to the treatment group being scored. Points are results from individual animals. Error bars represent SD. Data were analyzed by Student’s unpaired *t*-test. In this experiment, there were *N* = 4 animals in each group.

**Figure 3:**
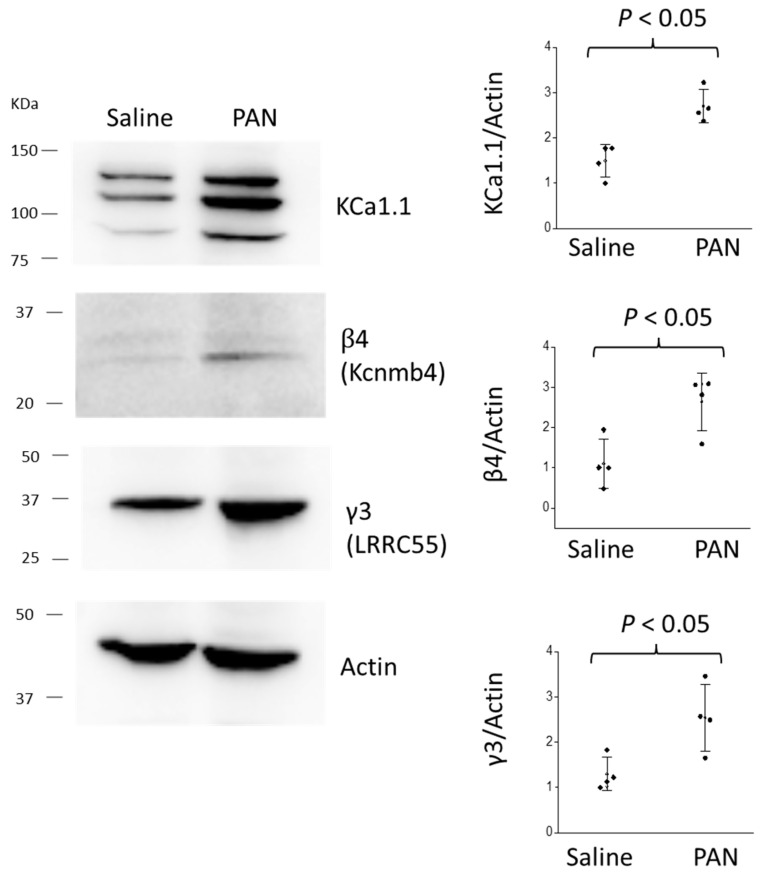
Increase in the expression of pore-forming and auxiliary subunits of BK channels in whole glomeruli isolated from saline- and PAN-treated rats during chronic PAN nephrosis. Representative immunoblots measuring pore-forming KCa1.1 subunits and auxiliary β4 and γ3 subunits are shown on the left (all from the same animal); quantitative densitometric analyses from the two groups of animals are shown on the right. Graphs show individual values and SD for *N* = 4 animals in each group.

We next examined the pattern of glomerular BK channel subunit expression in the chronic PAN model of FSGS in Sprague Dawley rats [6]. This model of nephrosis has been widely used since the 1980s [29]. In rats, PAN is selectively taken up by podocytes, where it is metabolized to produce a large quantity of reactive oxygen species, which rapidly cause the loss of a certain percentage of podocytes accompanied by substantial proteinuria that peaks during the first ~10 days following the treatment. This is referred to as the acute phase of PAN nephrosis and it reflects an acute toxic response to PAN. During the acute phase, one may see podocyte foot-process effacement but minimal or no changes at the light microscopic level [29]. The proteinuria subsequently subsides, but gradually returns over the next several weeks, a stage that is referred to as the chronic phase of PAN nephrosis. Glomerulosclerosis readily detectable at the light microscopic level accompanies this gradual recurrence of albuminuria, following the formation of adhesions between the glomerular basement membrane and the parietal layer [29]. The chronic phase of PAN nephrosis is, therefore, the relevant disease model for FSGS. In our experiments, Sprague Dawley rats were given a single injection of PAN, or a saline vehicle. The animals were euthanized 30 days later and the kidneys were removed for analysis. We have previously shown that complete TRPC6 inactivation exerts a significant protective effect in this model [6]. PAN-treated rats exhibited a significant increase in urine albumin excretion (Figure 2a) and serum creatinine (Figure 2b), as well as the marked glomerulosclerosis easily seen in the PAS-stained sections (Figure 2c,d). Glomeruli were isolated from these animals and the expression of BK channel subunits was examined using immunoblot methods. In those experiments, we observed significant increases in the overall abundance of KCa1.1, β4, and γ3 subunits in the PAN-treated animals compared to the saline-treated controls (Figure 3).

FSGS is not a single-disease entity, rather it is a clinical and histopathological pattern with a variety of etiologies, often classified as primary, secondary, or genetic, but which generally entails an initial injury to the podocytes. The chronic PAN model is considered a model for secondary FSGS, as it develops gradually over time following the PAN-evoked death of a certain percentage of podocytes. By contrast, primary FSGS is caused by endogenous circulating factors related to innate and adaptive immunity, and which often recurs in patients who have received a kidney graft [30].

**Figure 4:**
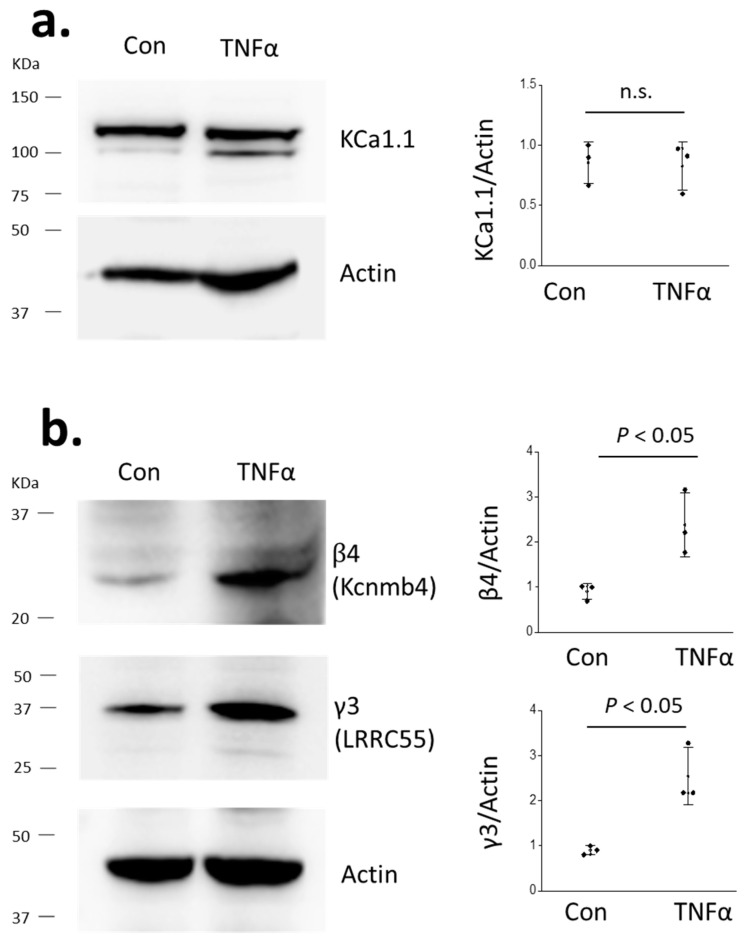
Effects of TNFα on BK channel subunits. Exposing cultured podocytes to 10 ng/mL TNFα for 24 h does not increase overall abundance of KCa1.1 (**a**) but increases overall abundance of auxiliary β4 and γ3 subunits (**b**). Representative blots are shown to the left. Points in graphs on the right are the results from each repetition and error bars represent SD. Data were analyzed by Student’s unpaired *t*-test.

Unfortunately, there are few, if any, reliable animal models for primary FSGS. The current status of circulating factors in primary FSGS has been reviewed recently [31]; the list of potential glomerular “permeability” factors includes suPAR, TNFα, IL-13, cardiotrophin-like cytokine factor-1, B-cell activating factor, hemopexin, and possibly others. In the present study, we examined the effects of suPAR and TNFα because our prior studies have shown that both are able to markedly increase TRPC6 surface expression and activity in podocytes [25,26]. Here, we observed that the 24 h exposure of podocytes to a medium containing 10 ng/mL TNFα resulted in an increase in the overall abundance of β4 and γ3, but not of KCa1.1 (Figure 4).

**Figure 5:**
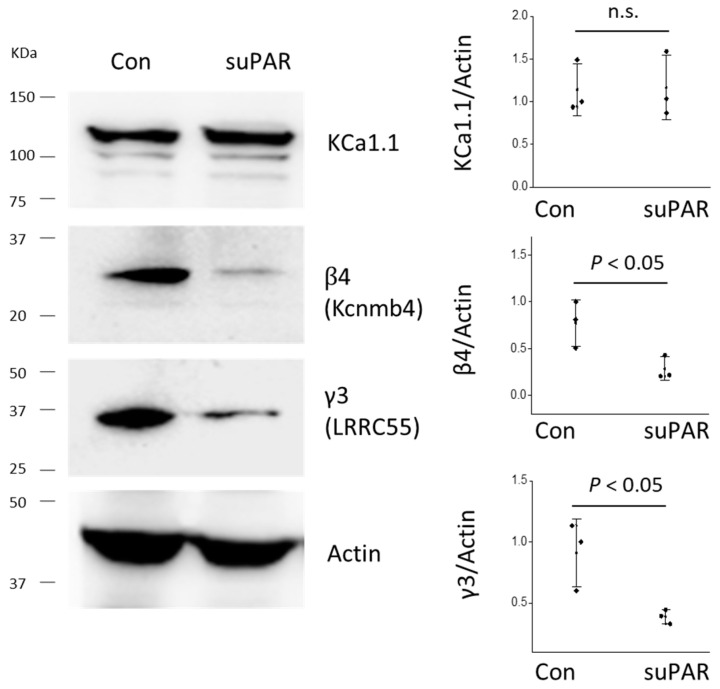
Exposing cultured podocytes to 20 ng/mL suPAR for 24 h had no effect on the overall abundance of pore-forming KCa1.1 subunits and caused a decrease in auxiliary β4 and γ3. Data were analyzed by Student’s unpaired *t*-test. n.s. denotes statistically non-significant difference.

By contrast, 24 h exposure to 20 ng/mL suPAR had no effect on the abundance of KCa1.1 subunits, and actually evoked a decrease in the abundance of β4 and γ3 (Figure 5).

**Figure 6:**
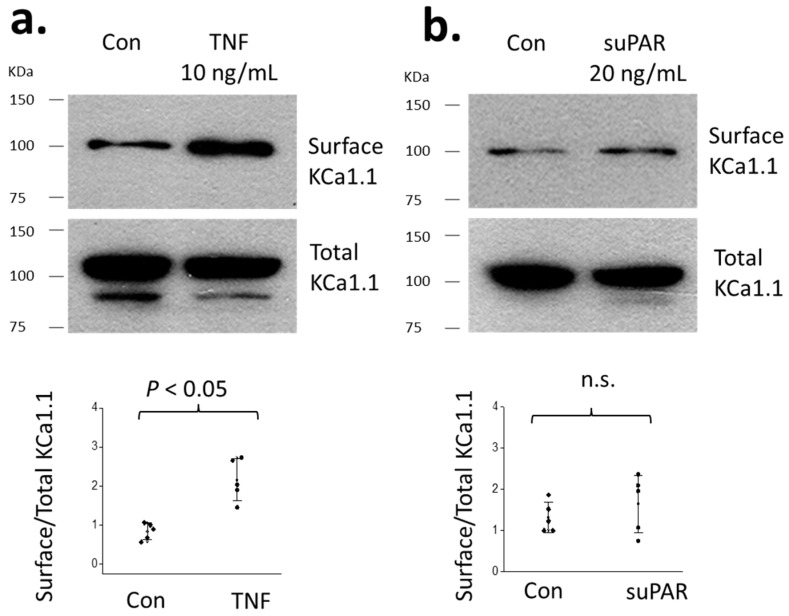
Exposing cultured podocytes to TNFα for 24 h increased cell surface expression of KCa1.1 (**a**); whereas suPAR had no effect (**b**). Graphs show individual values of the relative expression of surface and total KCa1.1 based on cell-surface biotinylation assays. Error bars represent SD. Data were analyzed by Student’s unpaired *t*-test. n.s. denotes statistically non-significant difference.

KCa1.1 trafficking to the podocyte cell surface is a highly regulated process [19]. Therefore, we utilized cell-surface biotinylation assays to examine the effects of TNFα and suPAR on the steady-state surface expression of KCa1.1 in cultured mouse podocytes. We observed that TNFα evoked a marked increase in the surface expression of KCa1.1 compared to the control, whereas suPAR had no effect (Figure 6).

**Figure 7:**
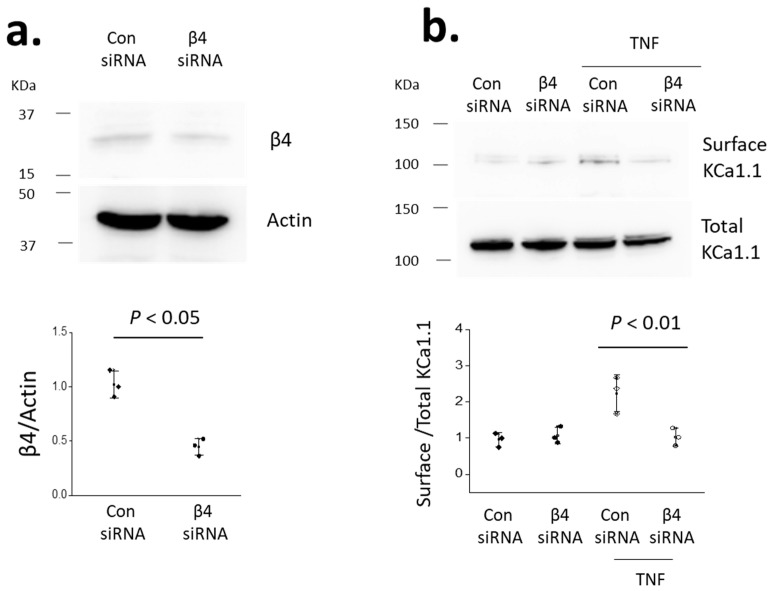
The stimulatory effect of TNFα on the surface expression of KCa1.1 subunits in podocytes was inhibited by siRNA knockdown of β4 subunits: (**a**) immunoblot analysis showing reduced expression of the β4 subunit by the siRNA targeting that subunit compared to the control siRNA; and (**b**) representative cell-surface biotinylation assays are shown above the quantitative analyses of these types of experiments. There was a significant difference in the groups by one-way ANOVA (*F* = 11.05, *p* < 0.01); the siRNA targeting the β4 subunit significantly reduced the effects of TNFα (*p* < 0.01, Tukey’s honestly significant difference post hoc test).

We have previously shown that many of the KCa1.1 subunits endogenously expressed in mouse podocytes are VEDEC variants, which refers to the last five residues at the carboxy terminus [18,19]. KCa1.1 subunits containing this motif are generally retained in intracellular compartments in a variety of cell types, but traffic to the surface in response to certain hormones [32] and as a result of interactions with other proteins, including β1-subunits of BK channels [33]. In addition, the VEDEC variants evoke a dominant-negative effect on the surface expression of other KCa1.1 splice variants [18]. In the present study, we have focused on the β4 subunit, which is the main β subunit expressed in podocytes [19]. We hypothesized that the effects of TNFα on the surface expression of KCa1.1 would correlate with their effects on the overall abundance of β4 subunits. This hypothesis predicts that suppressing the expression of β4 should reduce the stimulatory effects of TNFα on the surface abundance of KCa1.1. To test this hypothesis, we utilized siRNA techniques to reduce the abundance of β4 subunits in cultured podocytes; we measured the surface expression of KCa1.1 following 24 h exposure to 10 ng/mL TNFα (Figure 7). We observed that knockdown of the β4 subunit eliminated the ability of TNFα to stimulate surface expression of KCa1.1.

## 4. Discussion

Sustained, excessive Ca^2+^ influx into podocytes contributes to the progression of chronic kidney disease [1]. Podocytes are non-excitable cells that, by definition, lack the voltage-gated Ca^2+^ channels found in nerves and muscles. Instead, non-selective cation channels, including TRPC channels, represent the most important sources of Ca^2+^ influx. However, the permeation properties of TRPC6 place significant limits on the conditions wherein these particular channels can sustain Ca^2+^ influx [16]. Specifically, a site within the TRPC6 pore binds Ca^2+^ with an affinity that depends on the trans-membrane electric field, which affects ion permeation through the pore. As a result, when the cells are depolarized, TRPC6 channels on the cell surface function as monovalent cation channels and become essentially impermeable to Ca^2+^ [16]. The activation of any type of TRPC channel invariably causes membrane depolarization in physiological conditions; this occurs regardless of whether or not divalent cations are able to permeate the pore. Because of these inherent biophysical properties, TRPC6, and probably other TRPC channels, cannot be a significant source of Ca^2+^ influx unless there is a mechanism in place to limit the cell depolarization caused by their own activity. Put another way, TRPC6 channels effectively have a voltage dependence that is the opposite of voltage-gated Ca^2+^ channels, although this is a consequence of the permeation properties of the pores rather than gating of the channels, per se.

We have previously shown that the native BK channels of podocytes immunoprecipitate with TRPC6 [15]. This ensures that the Ca^2+^-binding domains of KCa1.1 subunits lie within a zone of elevated Ca^2+^ immediately surrounding the active TRPC6 channels, similar to the co-localization of KCa.1 with voltage-gated Ca^2+^ channels seen in excitable cells [34]. Moreover, currents through the BK channels of podocytes are markedly reduced under conditions where the distance that Ca^2+^ can diffuse away from a TRPC6 pore is reduced, or following TRPC6 knockdown [1]. In the present study, we have confirmed that the pharmacological inhibition of BK channels markedly reduces Ca^2+^ influx evoked by a stimulus that induces TRPC6 activation. We should note that this particular result confirms the earlier observations of Hu et al. [22], who used similar imaging techniques in podocytes to reach the same conclusion (although it should be noted that those workers used a different TRPC6-activating stimulus). In other words, the coupling of BK channels to TRPC6 activation helps to sustain Ca^2+^ influx; therefore, it is possible that they contribute to Ca^2+^ overload in podocytes, especially if BK channels are upregulated in podocyte disease.

Here, we also observed that the total abundance of KCa1.1, β4, and γ3 subunits were all increased in glomeruli isolated from rats during the chronic phase of PAN nephrosis. This model has long been known to induce glomerulosclerosis and nephrosis, as well as effects secondary to the podocyte injury in nephrons and the cortical interstitium [29]. It is notable that all three classes of BK subunits were increased in this model. In this regard, an increase in γ3 subunits has been previously reported in mice treated with doxorubicin and, significantly, in humans with nephrotic syndromes [22]. BK channels function essentially as voltage-activated K^+^ channels, in which Ca^2+^-binding induces a marked left-shift in voltage dependence through strong allosteric effects [35]. β4 subunits cause a large left-shift in the voltage dependence of BK activation to well within the physiological range of membrane potentials for podocytes [17]. β4 subunits also cause marked slowing of BK activation and confer partial resistance to certain venom toxins, such as iberiotoxin, both of which are features of the native BK channels of podocytes [19]. γ3 subunits also lead to enhanced BK activation through marked left-shifts in voltage dependence [17,36].

Podocytes express VEDEC variants of KCa1.1 subunits, which refers to the last five residues in the carboxy terminus [18,19]. The VEDEC variants tend to be retained in intracellular compartments and exert a dominant-negative effect on the surface expression of other carboxy-terminal KCa1.1 splice variants [18]. The steady-state expression of VEDEC variants on the cell surface requires signals from growth factors or hormones (e.g., insulin [32]) and/or interactions with other proteins, including BK channel β1 subunits in neurons [33] and certain slit-diaphragm proteins in the case of podocytes [19]. Through this effect, increases in the expression of β4 subunits could lead to increases in the surface expression of KCa1.1 subunits in podocytes. For that reason, we also examined the steady-state surface expression of KCa1.1 in vitro.

In those experiments, we examined TNFα and suPAR, two circulating factors that have been implicated in FSGS, and which we have previously shown to markedly enhance TRPC6 trafficking to the podocyte cell surface [25]. Neither of these factors evoked an increase in the overall abundance of KCa1.1; however, TNFα induced a significant increase in the total abundance of β4 and γ3 subunits. We were surprised to see that suPAR actually caused a decrease in the overall abundance of β4 and γ3. TNFα also evoked a marked increase in the surface expression of KCa1.1; however, suPAR had no effect on KCa1.1 trafficking. It is possible that the basal surface expression of KCa1.1 (in the absence of circulating factors) is due to EMVYR variants, which do not require auxiliary subunits for trafficking to the cell surface [18]. Finally, we observed that siRNA knockdown of the β4 subunit inhibited the effects of TNFα on the surface abundance of KCa1.1.

These results allow for certain conclusions. First, TNFα is sufficient to increase the cell surface expression of KCa1.1 subunits; this effect requires the presence of the β4 subunit. It should be noted that there are case reports of children with primary FSGS achieving remissions following treatment with TNFα inhibitors [37,38,39] and that TNFα pathway activation is a factor in a subset of FSGS and patients with minimal change disease with poor clinical outcomes [40]. Second, suPAR by itself cannot increase KCa1.1 at any concentration that we have tested, which is in marked contrast to its effect on TRPC6 [25,26]. This observation suggests that the increased trafficking of TRPC6 to the cell surface (which occurs in response to either suPAR or TNFα) does not carry the VEDEC variants of KCa1.1 along with it to the cell surface. Other mechanisms must be engaged, which probably include the increase in β4 subunits. However, KCa1.1 channels that reach the cell surface are able to form a complex with TRPC6 [15], which enables their coordinated activation [1].

Finally, these results are consistent with models of primary FSGS in which multiple circulating factors simultaneously contribute to the pathology, including various effects on podocyte cell biology, including Ca^2+^ regulation and its downstream pathways (e.g., calcineurin, calpain, etc.). In other words, in primary FSGS the circulation is likely to contain an entire milieu of factors related to innate and adaptive immunity, inflammation, and possibly other processes [31,41]. The implication of that model is that looking for a single factor that would explain primary FSGS in all patients is not going to succeed. It could also explain some of the controversies in the literature on suPAR and TNFα in the context of nephrosis [31]. On the other hand, targeting any of factors that are necessary for pathology in a given milieu could be a successful strategy, even if they are not measurably increased in some patients.

Finally, the question arises as to why circulating factors might regulate the ion channel dynamics in podocytes. It was suggested a long time ago that the glomerular filtration barrier adjusts its permselectivity to allow for proteinuria in response to infections and possibly in other conditions where there is an increase in damage or pathogen-related signals, including endotoxins [42]. In this regard, SARS-CoV-2 proteins have recently been shown to induce a rapid change in podocyte function that leads to proteinuria, which in certain conditions is mediated by suPAR [43]. Changing the function of ion channels that regulate foot process dynamics and podocyte gene expression may play a role in proteinuria that occurs during acute infections or in response to tissue damage. It is also possible that these signals can become maladaptive if they are excessively activated in amplitude or duration, as can occur in certain chronic inflammatory states. There are a number of mechanisms whereby multiple circulating factors can produce synergistic effects on the glomerular filtration barrier. Enhancing the surface expression and activity of K^+^ channels, such as KCa1.1, is a mechanism that could enhance Ca^2+^ overload caused by influx through non-selective cation channels such as TRPC6. We conclude here that this can be mediated by actions on the BK channel auxiliary subunits.

## Data Availability

All data are available upon request.
